# Draft Genome Sequence of the First Documented Clinical Siccibacter turicensis Isolate in Austria

**DOI:** 10.1128/genomeA.00380-18

**Published:** 2018-05-03

**Authors:** Sarah Lepuschitz, Shiva Pekard-Amenitsch, Renée Haunold, Simone Schill, Agnes Schriebl, Robert Mach, Franz Allerberger, Werner Ruppitsch, Stephen J. Forsythe

**Affiliations:** a Institute of Medical Microbiology and Hygiene, Austrian Agency for Health and Food Safety, Graz, Austria; b Research Area of Biochemical Technology, Institute of Chemical, Biological and Environmental Engineering, Vienna University of Technology, Vienna, Austria; c Labor Dr. Berset, Vienna, Austria; d Institute of Milk Hygiene, Milk Technology and Food Science, University of Veterinary Medicine, Vienna, Austria; e Department of Biotechnology, University of Natural Resources and Life Sciences, Vienna, Austria; f foodmicrobe.com, Adams Hill, Keyworth, United Kingdom

## Abstract

The nonpathogenic species Siccibacter turicensis is closely related to members of the food-associated pathogenic genus Cronobacter and has been detected in fruit powders, formula, spices, and herbs. Here, we report on the first clinical isolate of S. turicensis, recovered from the labial angle of a patient with angular cheilitis.

## GENOME ANNOUNCEMENT

Members of the genus Siccibacter and family Enterobacteriaceae are characterized to be Gram negative, coccoid to rod shaped, peritrichously flagellated, weakly oxidase positive, catalase positive, and facultatively anaerobic (1). As close relatives of the foodborne pathogenic members of the genus Cronobacter, Siccibacter species can cause severe clinical infections in infants and immunocompromised adults; thus, their correct identification is of utmost importance. To date, S. turicensis has been isolated from various foods but has not been described in clinical samples (2).

In April 2017, a 40-year-old patient with a 6-month history of perleche saw a dermatologist. A swab taken from her mouth angle grew two types of Gram-negative rods, which were initially diagnosed as Cronobacter sakazakii and Escherichia vulneris. Topical treatment with gentamicin resulted in the healing of this angular cheilitis. Whole-genome sequencing performed on the assumed Cronobacter isolate revealed it to be Siccibacter turicensis.

The isolation of high-molecular-weight DNA from a bacterial overnight culture was carried out with a MagAttract HMW DNA kit (Qiagen, Hilden, Germany) and quantified with a Qubit version 2.0 fluorometer (Thermo Fisher Scientific, Waltham, MA, USA) using the double-stranded DNA broad-range (dsDNA BR) assay kit (Thermo Fisher Scientific). A NexteraXT kit (Illumina, Inc., San Diego, CA, USA) was used for library preparation. Whole-genome sequencing was done with 300-bp paired-end reads on an Illumina MiSeq instrument using the MiSeq reagent kit with V3 chemistry (Illumina). The *de novo* genome assembly was completed using SPAdes version 3.9.0 (3) and resulted in 171 contigs with a total of 4,224,698 nucleotides and a 58.4% GC content. Species confirmation was done via ribosomal multilocus sequence typing (4), and following submission to the Cronobacter PubMLST database (5), we assigned a new sequence type (ST), ST635, to the isolate (*Cronobacter* PubMLST ID 2411). The NCBI Prokaryotic Genome Annotation Pipeline (PGAP) identified 4,169 genes, 4,059 coding sequences, 128 pseudogenes, 24 rRNA operons (9 complete, 15 partially), and 76 tRNAs. Antimicrobial resistance genes were identified via the Comprehensive Antibiotic Resistance Database (CARD) (6) and included the antibiotic efflux genes CRP, *emrB*, *emrR*, H-NS, *marA*, *marR*, *msbA*, and *patA*, as well as the antibiotic inactivation gene *fosA2* and the antibiotic target alteration gene *glpT*. *In vitro* susceptibility testing with the Vitek 2 compact system (bioMérieux, Marcy-l’Étoile, France)—interpreted according to the European Committee on Antimicrobial Susceptibility Testing (EUCAST) clinical breakpoint tables version 8.0 (valid from 1 January 2018)—revealed the isolate to be resistant to fosfomycin and sensitive to ampicillin, amoxicillin-clavulanic acid, piperacillin-tazobactam, cefuroxime-axetil, cefoxitin, cefotaxime, ceftazidime, cefepime, aztreonam, imipenem, meropenem, amikacin, gentamicin, ciprofloxacin, moxifloxacin, tigecycline, and trimethoprim-sulfamethoxazole.

### Accession number(s).

This whole-genome shotgun project has been deposited at DDBJ/ENA/GenBank under the accession number PYEP00000000. The version described in this paper is the first version, PYEP01000000.
